# Supplementary figures and images for: Variation and inheritance of the Xanthomonas raxX‐raxSTAB gene cluster required for activation of XA21‐mediated immunity
Source: Mol Plant Pathol. 2019 Feb 18;20(5):656–72. doi: 10.1111/mpp.12783 (PMC6637879; doi:10.1111/mpp.12783)

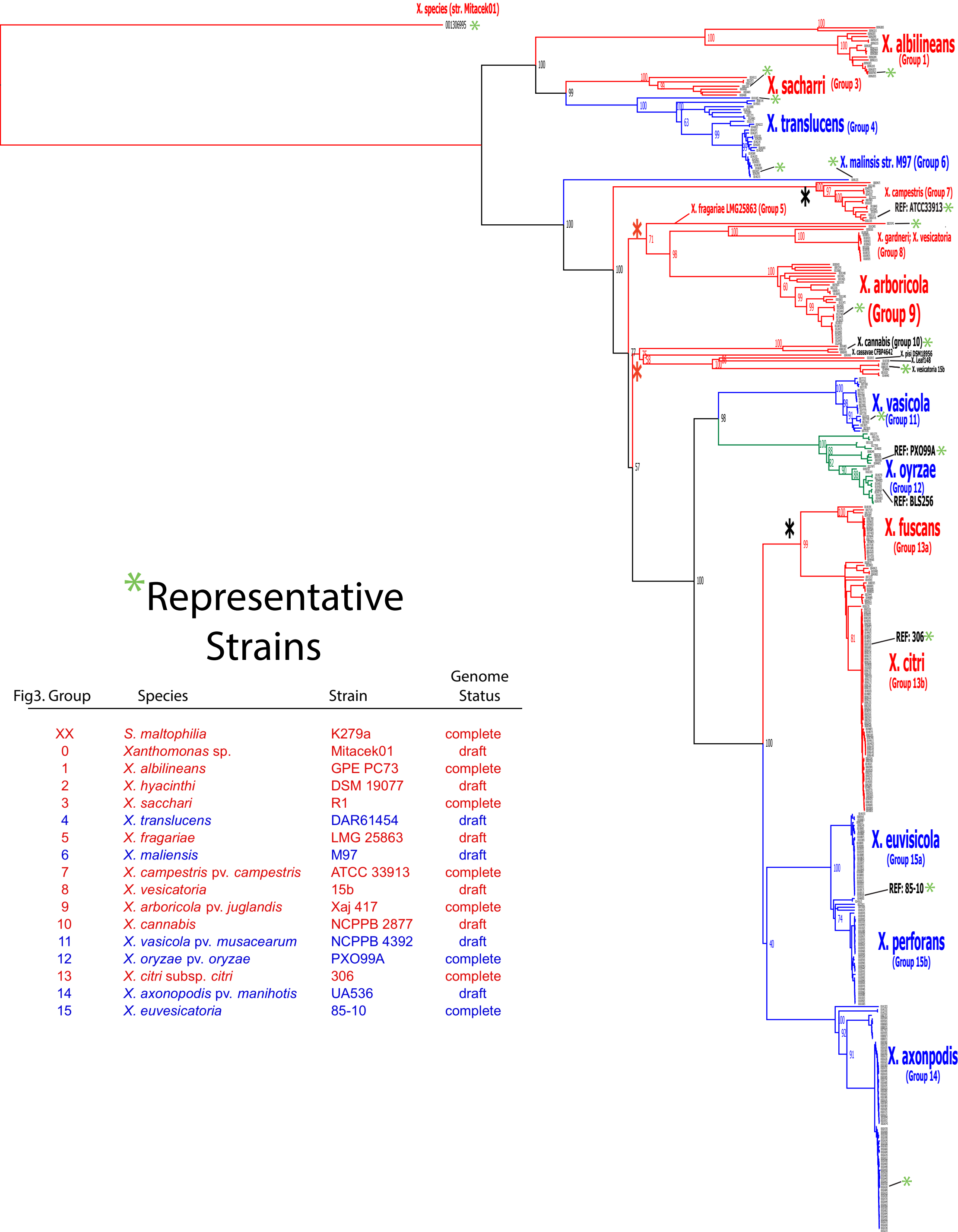

Supplement: Supplementary file 1 — Fig. S1 Whole‐genome‐based Xanthomonas phylogenetic tree. This tree was constructed by the analysis of whole‐genome sequences, as described in Experimental procedures. Blue indicates genomes that contain the raxX‐raxSTAB gene cluster; red indicates genomes that do not. Group numbers are arbitrary. [file MPP-20-656-s001.pdf]

## A. Left Boundary

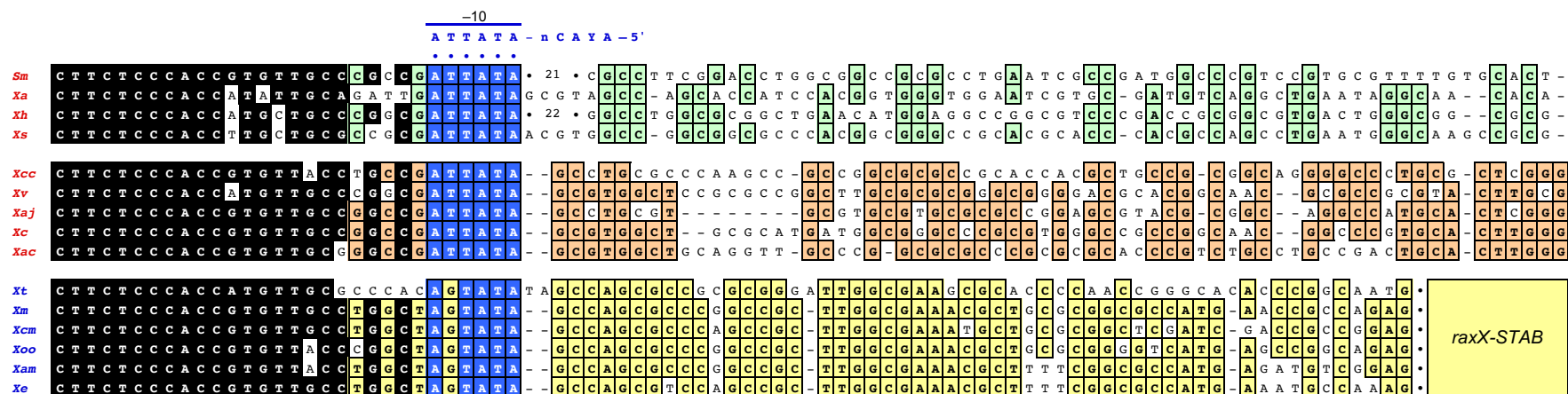

## B. Right Boundary

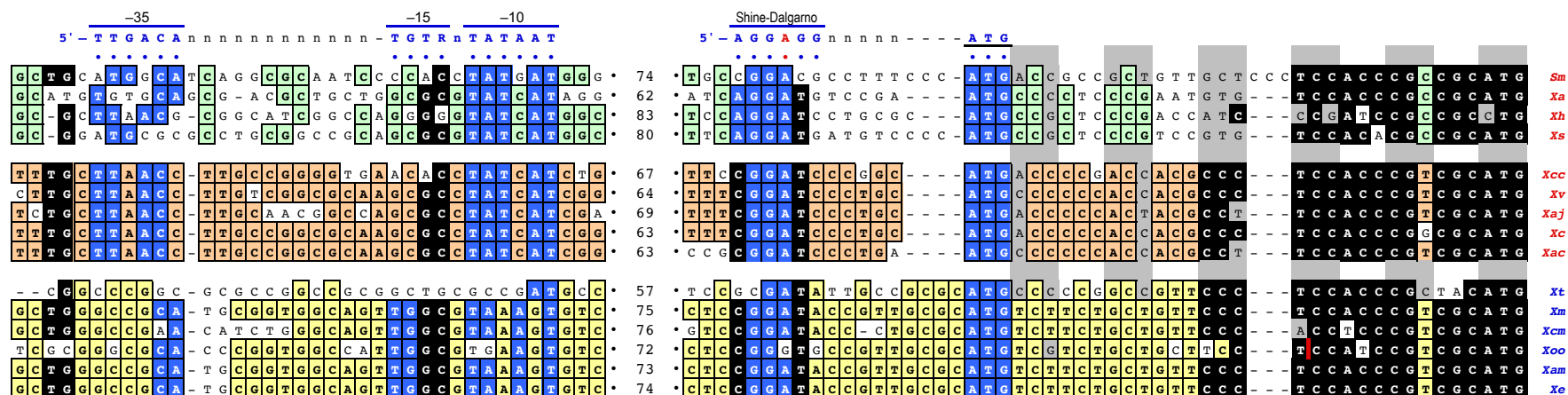

Supplement: Supplementary file 2 — Fig. S2 Sequences flanking the raxX‐raxSTAB gene cluster. Sequences are from the reference strains described in Table 1. Sequences conserved within a group, but different from other groups, are coloured green (‘early‐branching’ species), brown (raxX‐raxSTAB cluster‐negative strains) or yellow (raxX‐raxSTAB cluster‐positive strains). For presentation, the sequence is divided into left and right boundaries. The green and brown sequences are contiguous, whereas the yellow sequences are interrupted by the c. 5‐kb raxX‐raxSTAB gene cluster, depicted as a yellow rectangle. For presentation, approximately 60–80 nucleotides with relatively low similarity were removed from the sequence shown in the right boundary panel. These conceptual deletions are denoted by the number of nucleotides removed in each case. Black sequences are conserved in all lineages, and include both coding regions as well as matches to transcription and translation initiation consensus sequences, which are described in the text. An ‘mfsX’ + 1 frameshift in Xoo sequences is indicated by the vertical red line. Abbreviations are in red for raxX‐raxSTAB cluster‐negative strains and in blue for raxX‐raxSTAB cluster‐positive strains: Sm, Stenotrophomonas maltophilia; Xa, Xanthomonas albilineans; Xac, X. citri ssp. citri; Xaj, X. arboricola pv. juglandis; Xam, X. axonopodis pv. manihotis; Xc, X. cannabis; Xcc, X. campestris pv. campestris; Xcm, X. campestris pv. musacearum; Xe, X. euvesicatoria; Xf, X. fragariae; Xh, X. hyacinthi; Xm, X. maliensis; Xoo, X. oryzae pv. oryzae; Xs, X. sacchari; Xt, X. translucens; Xv, X. vesicatoria. [file MPP-20-656-s002.pdf]

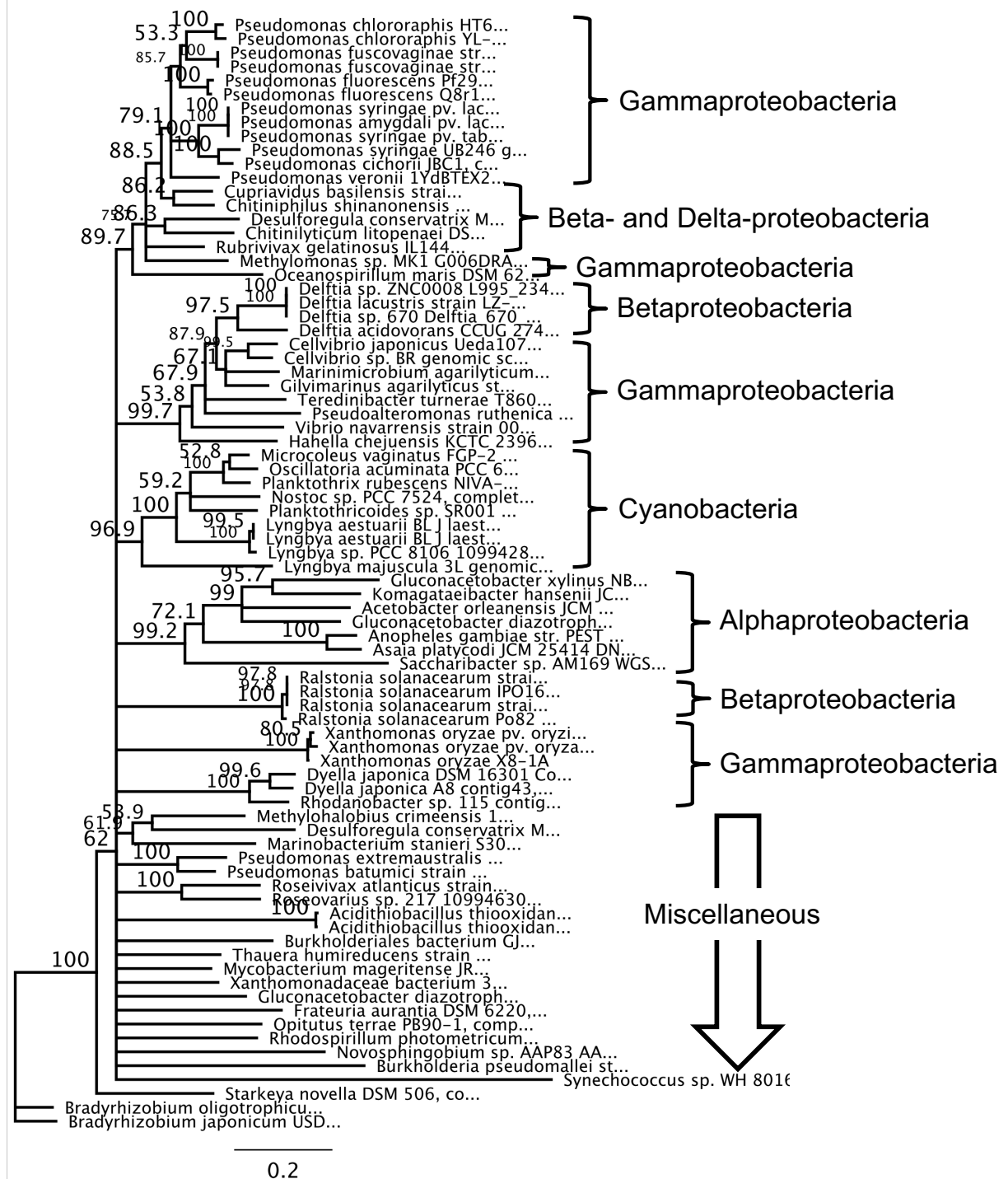

Supplement: Supplementary file 4 — Fig. S4 Phylogenetic tree for raxST homologues. Distribution of raxST homologues across bacterial genera, including the major groups of proteobacteria as well as cyanobacteria. The tree shown was constructed by neighbour‐joining with 1000 bootstrap replicates; branches with <50% bootstrap support are not drawn. The raxST sequence from Xanthomonas oryzae pv. oryzae (Xoo) strain PXO99A was used as query for tblastn. [file MPP-20-656-s004.pdf]

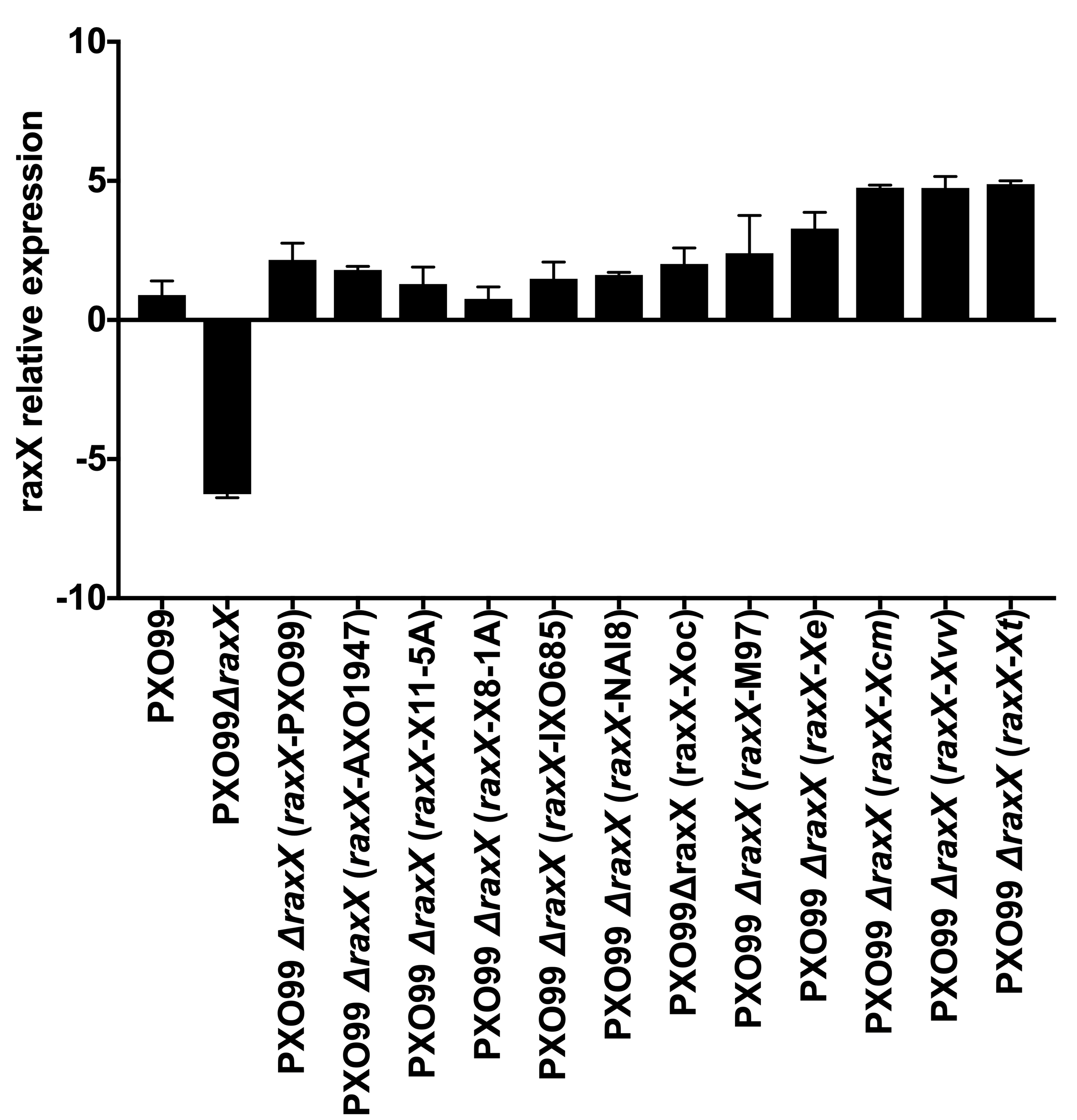

Supplement: Supplementary file 5 — Fig. S5 raxX expression in Xanthomonas oryzae pv. oryzae (Xoo) PXO99A complemented strains. Data show raxX gene expression in the complemented strains with different raxX alleles with its promoter region on plasmids. The expression is shown as the logarithm of raw data using quantitative reverse transcription‐polymerase chain reaction (qRT‐PCR). Data are the mean values from two biological replicates. Error bars show the standard deviation. [file MPP-20-656-s005.tiff]

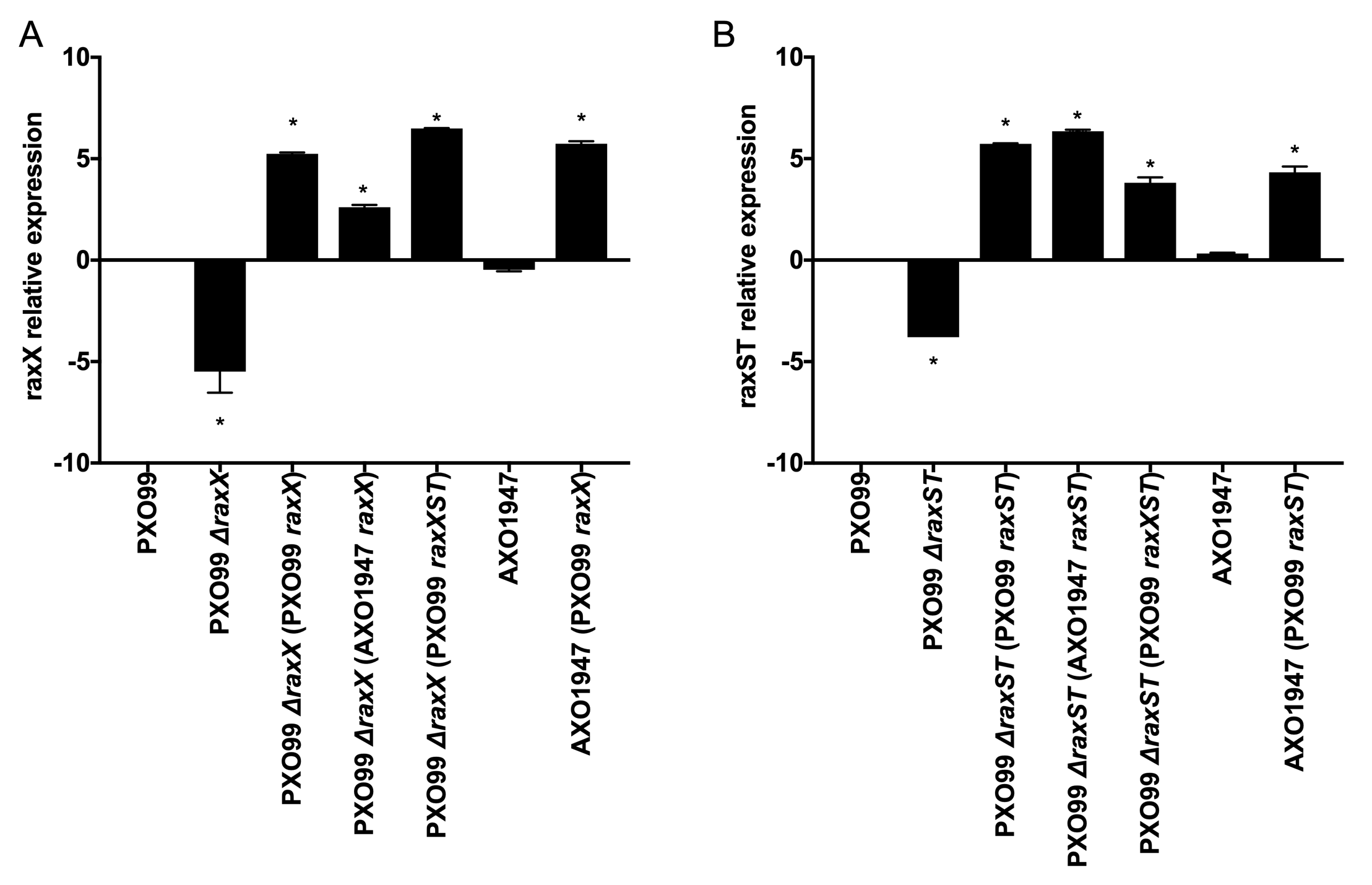

Supplement: Supplementary file 7 — Fig. S7 raxX and raxST expression in Xanthomonas oryzae pv. oryzae (Xoo) PXO99A complemented strains. Data show raxX and raxST gene expression in the complemented strains (with raxX and raxST on plasmids) relative to expression in Xoo strain PXO899A (with raxX and raxST on the chromosome). Expression was determined by quantitative reverse transcription‐polymerase chain reaction (qRT‐PCR) (see Experimental procedures), and is shown as the logarithm of the fold change. Gene expression was normalized to the chromosomal gene PXO_01660 (annotated as an ampC gene homologue encoding‐lactamase). Data are the mean values from two biological replicates. Error bars show the standard deviation. [file MPP-20-656-s007.tiff]

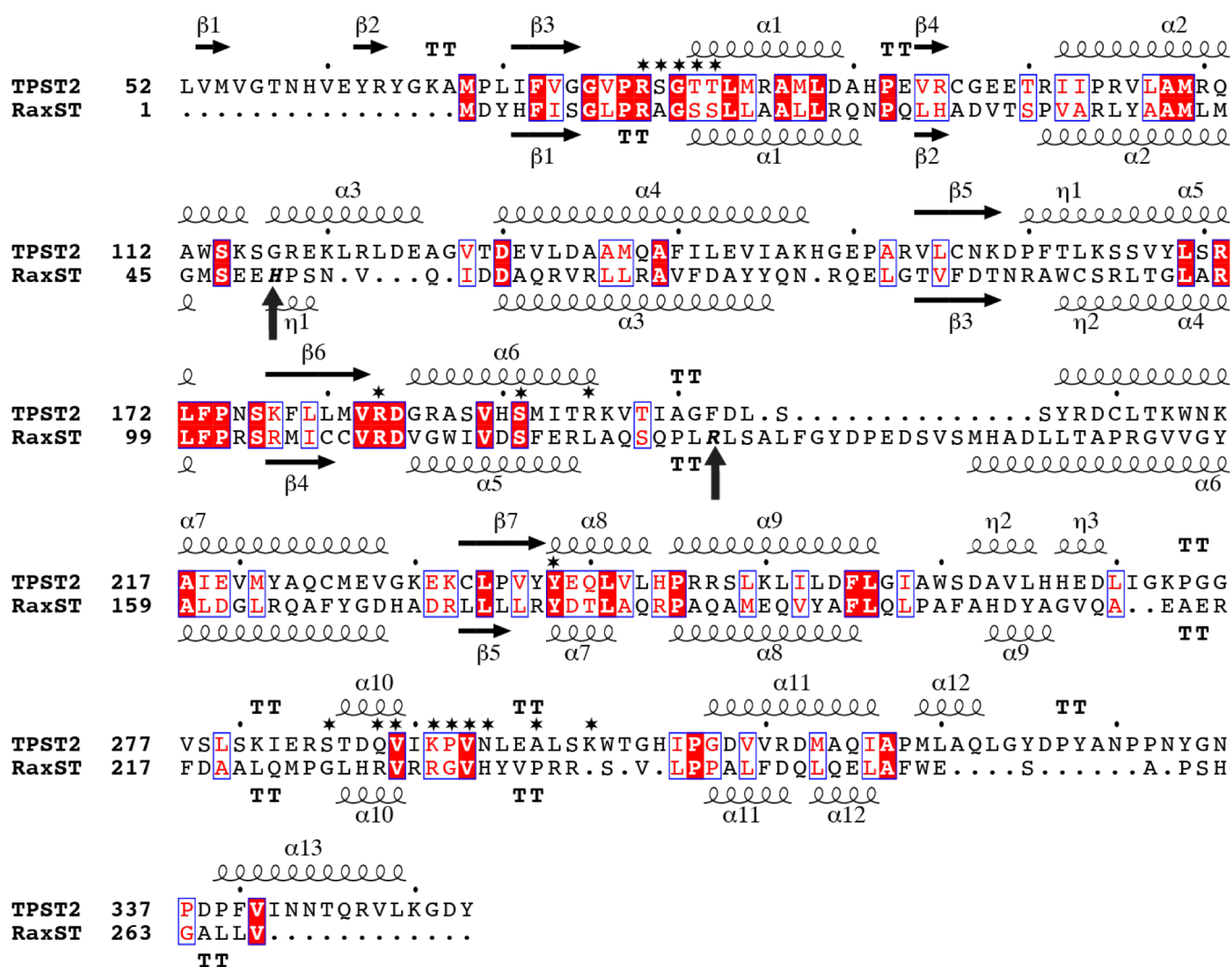

Supplement: Supplementary file 8 — Fig. S8 RaxST structural alignment. Sequence alignment of the human tyrosylprotein sulfotransferase‐2 (TPST2) and Xanthomonas oryzae pv. oryzae (Xoo) RaxST sequences formatted with ESPript 3.0 (Robert & Gouet, 2014). Secondary structure elements derived from the respective structural models are shown. Stars show TPST2 residues involved in 3′‐phosphoadenosine 5′‐phosphosulfate (PAPS) binding, and arrows show RaxST missense substitutions. [file MPP-20-656-s008.pdf]

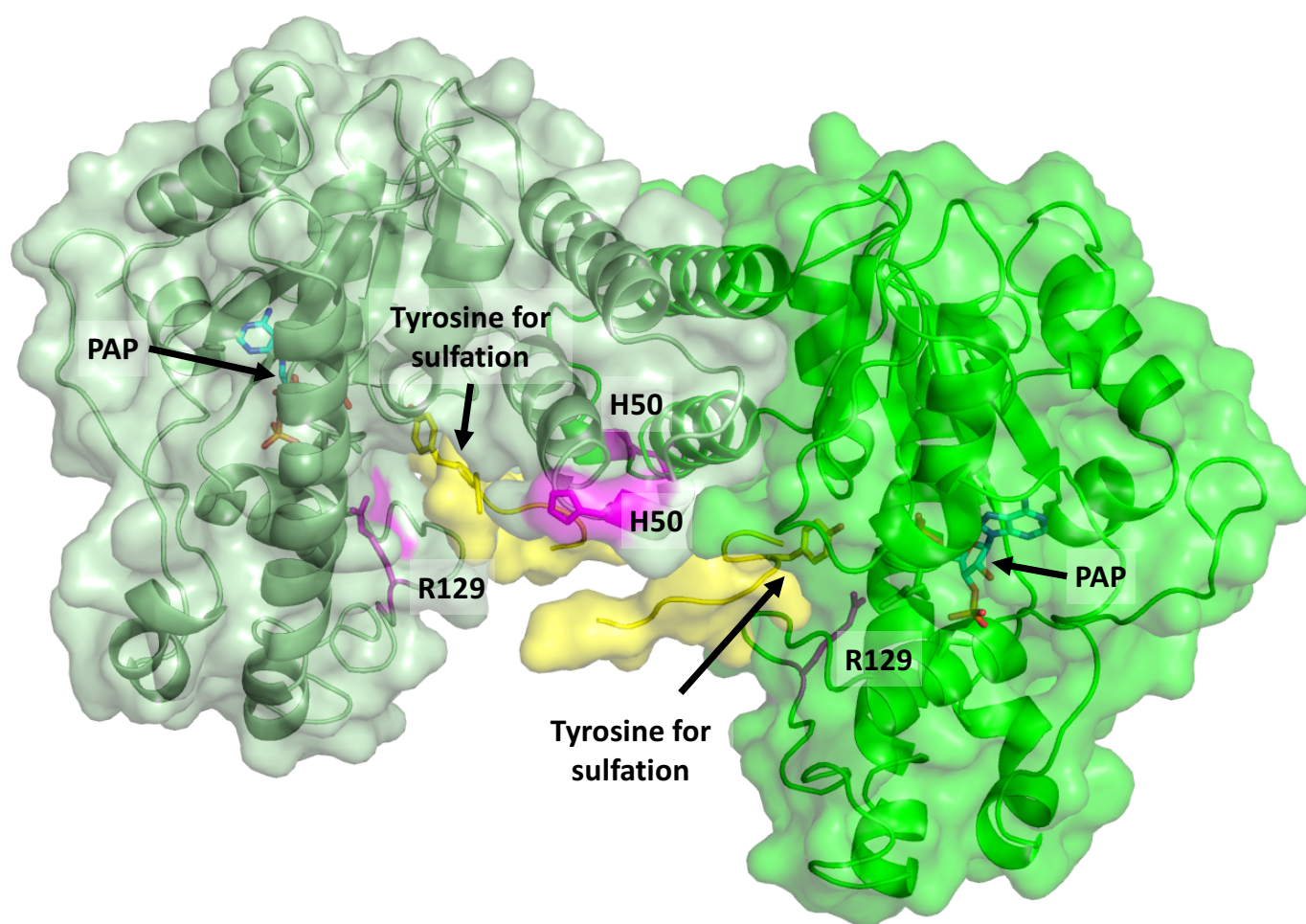

Supplement: Supplementary file 9 — Fig. S9 Model for RaxST structure. Predicted RaxST structure shown in cartoon and surface representation, based on the dimeric structure of tyrosylprotein sulfotransferase‐2 (TPST2). The two RaxST monomers are coloured in dark and light green. The 3′‐phosphoadenosine 5′‐phosphate (PAP) and C4 substrate peptide that were co‐crystallized with TPST2 are superimposed on the RaxST model. PAP is represented as labelled and the substrate peptide is shown in yellow cartoon with the acceptor tyrosine represented as labelled. Residues His‐50 and Arg‐129 are coloured in magenta and highlighted. [file MPP-20-656-s009.pdf]
