# Supplementary material for: Variation and inheritance of the Xanthomonas raxX‐raxSTAB gene cluster required for activation of XA21‐mediated immunity
Source: Mol Plant Pathol. 2019 Feb 18;20(5):656–72. doi: 10.1111/mpp.12783 (PMC6637879; doi:10.1111/mpp.12783)
Supplement: Supplementary file 3 — Fig. S3 GcvP length polymorphisms in different Xanthomonas lineages. The relevant portion of the GcvP amino acid sequence is shown for each of the reference strains. Species in red lack the raxX‐raxSTAB gene cluster, whereas those in blue carry the cluster. Numbers denote different allelic types for reference to Fig. 3. The positions of residues Gly‐733 and Val‐738 (numbering for allelic type 1) are indicated. Abbreviations: Sm, Stenotrophomonas maltophilia; Xa, Xanthomonas albilineans; Xac, X. citri ssp. citri; Xaj, X. arboricola pv. juglandis; Xam, X. axonopodis pv. manihotis; Xc, X. cannabis; Xcc, X. campestris pv. campestris; Xcm, X. campestris pv. musacearum; Xe, X. euvesicatoria; Xf, X. fragariae; Xh, X. hyacinthi; Xm, X. maliensis; Xoo, X. oryzae pv. oryzae; Xs, X. sacchari; Xt, X. translucens; Xv, X. vesicatoria. [file MPP-20-656-s003.pdf]

```
Xaj  2a   GVGPCAVKSHLAPYLPRAGI----HAGEGQTAAIHGGGLNSESGSGHSSRIGGMVSAAAYGSASILPISWM
Xcm  2a   GVGPCAVKSHLAPYLPRAGI----HAGEGQDVAAHGGGLNSESGAAGSLRTGGMVSAAAYGSASILPISWM
Xoo  2a   GVGPCAVKSHLAPFLPRAGL----HAGEGQTAAIHGGGFNSGSGSGHSSRIGGMVSAAAYGSASILPISWM
Xac  2a   GVGPCAVKSHLAPYLPRAGI----HAGEGQTAAIHGGGFNSESGNGHSSRIGGMVSAAAYGSASILPISWM
Xm   2b   GVGPCAVKSHLAPYLPRAGI--------------HGGGFNSESGSGHSSRIGGMVSAAAYGSASILPISWM
Xoc  2b   GVGPCAVKSHLAPFLPRAGL--------------HAGGFNSESGSGHSSRIGGMVSAAAYGSASILPISWM
Xcc  3a   GVGPCAVKSHLAPFLPKTLPNAGIRAGENQKAAIHGSGSNF--GEGE----VGMVSAASYGSASILPISWM
Xa   3b   GVGPCAVKAHLAPYLPMTLPN----AGEAQKAA----------GEGV----VGMVSAASFGSASILPISWM
Sm   1    GVGPCAVKEHLAPFLPGKLG-----------------------DNGP----VGMVSAASFGSASILPISWM
Xh   1    GVGPCAVKSHLAPYLPKTLG-----------------------GEGD----VGMVSAASFGSASILPISWM
Xs   1    GVGPCAVKAHLAPYLPKTLG-----------------------GDGE----VGMVSAASFGSASILPISWM
Xt   1    GVGPCAVKSHLAPYLPKTLG-----------------------GEGD----VGMVSAASFGSASILPISWM
Xf   1    GVGPCAVKSHLAPFLPRTLG-----------------------SEGD----VGMVSAASYGSASILPISWM
Xv   1    GVGPCAVKSHLAPFLPKTLG-----------------------GEGD----VGMVSAASYGSASILPISWM
Xc   1    GVGPCAVKSHLAPFLPRTLG-----------------------GEGD----VGMVSAASYGSASILPISWM
Xam  1    GVGPCAVKSHLAPFLPRTLG-----------------------GEGD----VGMVSAASYGSASILPISWM
Xe   1    GVGPCAVKSHLAPYLPKTLG-----------------------GEGD----VGMVSAASYGSASILPISWM
                                 ↑                               ↑
                              Gly-733                         Val-738
```
