# Supplementary material for: Variation and inheritance of the Xanthomonas raxX‐raxSTAB gene cluster required for activation of XA21‐mediated immunity
Source: Mol Plant Pathol. 2019 Feb 18;20(5):656–72. doi: 10.1111/mpp.12783 (PMC6637879; doi:10.1111/mpp.12783)
Supplement: Supplementary file 6 — Fig. S6 RaxST sequence polymorphisms in Xanthomonas oryzae pv. oryzae (Xoo) strain AXO1947. The RaxST sequence from Xoo strain PXO99A is shown. The seven missense substitutions in the sequence from Xoo strain AXO1947 (Huguet‐Tapia et al., 2016) are indicated. The boundaries of the 3′‐phosphoadenosine 5′‐phosphosulfate (PAPS) binding motifs (5′‐PSB and 3′‐PB; Negishi et al., 2001), enclosed in boxes, include the catalytic residues Arg‐11 and Ser‐118. [file MPP-20-656-s006.pdf]

```
          11                                      50
          •                                       •
VDYHFISGLPRAGSSLLAALLRQNPQLHADVTSPVARLYAAMLMGMSEEHPSNVQIDDAQ  60
         5'-PSB                                   D

              75                                          118
              •                                           •
RVRLLRAVFDAYYQNRQELGTVFDTNRAWCSRLTGLARLFPRSRMICCVRDVGWIVDSFE  120
              D                                    3'-PB

        129             145
        •               •
RLAQSQPLRLSALFGYDPEDSVSMHADLLTAPRGVVGYALDGLRQAFYGDHADRLLLLRY  180
        L               R

                     202
                     •
DTLAQRPAQAMEQVYAFLQLPAFAHDYAGVQAEAERFDAALQMPGLHRVRRGVHYVPRRS  240
                     T

        249               267
        •                 •
VLPPALFDQLQELAFWESAPSHGALLV                                   267
        R                 I
```
